# A patient with dialysis-dependent acute kidney injury due to hantavirus complicated with SARS-CoV-2 infection 

**DOI:** 10.5414/CNCS110846

**Published:** 2022-08-04

**Authors:** Virginia Geladari, Pantelis A. Sarafidis, Maria-Eleni Alexandrou, Danai Faitatzidou, Christina Nikolaidou, Maria Stangou, Aikaterini Papagianni

**Affiliations:** 1 Department of Nephrology, and; 2 Department of Pathology, Hippokration Hospital, Aristotle University of Thessaloniki, Thessaloniki, Greece

**Keywords:** acute kidney injury, acute interstitial nephritis, hantavirus nephropathy

## Abstract

In this case, we report a 64-year-old man presenting with anorexia, nausea and vomiting, mild abdominal pain, and oligoanuria for a few hours. His previous medical history included diabetes, hypertension, and chronic kidney disease (CKD) stage 3. Upon arrival, laboratory results revealed stage III acute kidney injury (AKI) with hyperkalemia requiring dialysis treatment. During hospitalization, both pre-renal and post-renal causes of AKI were excluded, and a careful diagnostic evaluation, including kidney biopsy and serology testing, revealed acute interstitial nephritis and positive IgM for hantavirus. The patient was started on steroid treatment, which led to complete recovery of kidney function over 3 months. Moreover, during his hospitalization, the patient was also diagnosed with SARS-CoV-2 infection, possibly due to intra-hospital transmission and was hospitalized at the COVID-19 Department for 14 days, eventually with no further complications. Hantavirus nephropathy should be at the differential diagnosis of AKI, even in the absence of typical symptoms. Steroid treatment may be helpful in reversal of kidney injury.

## Introduction 

Hantaan hemorrhagic fever is a rare zoonosis caused by hantaviruses of the Bunyavirales order; it is transmitted by rodents and represents an uncommon cause of acute kidney injury (AKI). Hantavirus infections cause two clinical syndromes in humans: hemorrhagic fever with renal syndrome (HFRS) in Asia and Europe, and hantavirus pulmonary (or cardiopulmonary) syndrome (HPS) in the Americas, with fatality rates of up to 12 and 60%, respectively [1]. HFRS is characterized by fever and flu-like symptoms, AKI with oligoanuria, pyuria, severe thrombocytopenia, left-shifted leukocytosis, anemia, and coagulation dysfunction mimicking thrombotic microangiopathies, which may progress to hemorrhagic shock [2]. Depending on the virus strain that is endemic in different geographic locations, the clinical presentation ranges from mild to severe [2]. 

Herein, we describe a patient presenting with oligoanuric, dialysis-dependent AKI, without hemorrhagic manifestations. A biopsy was performed revealing acute interstitial nephritis (AIN), while further immunologic evaluation confirmed recent infection with hantavirus. Furthermore, the patient was infected with SARS-CoV-2 during his hospitalization, possibly due to intra-hospital transmission. Despite the above, full recovery of patient’s kidney function was achieved with appropriate treatment. 

## Case report 

### Clinical presentation 

A 64-year-old Caucasian man presented to the Emergency Department of our tertiary hospital reporting oligoanuria, anorexia, nausea, and vomiting for the past 4 days, together with diffuse abdominal pain for the last 12 hours. He was a farmer from a northern Greek village. He reported no history of fever, macroscopic hematuria, or gastrointestinal bleeding. His past medical history included diabetes and essential hypertension during the last 15 years, bilateral carotid stenosis (right 60% and left 43%), benign prostate hyperplasia, and an inguinal hernia surgery. His daily medication consisted of metformin 1,000 mg once daily (o.d.), a combination of olmesartan 10 mg/amlodipine 2.5 mg/hydrochlorothiazide 6.25 mg o.d., acetylsalicylic acid 100 mg o.d., atorvastatin 20 mg o.d. and finasteride 5 mg o.d. No other drug had been recently initiated or received sporadically by the patient. Past laboratory exams also revealed the presence of chronic kidney disease (CKD) with serum creatinine (SCr) of 1.29 mg/dL 1.5 years and SCr of 1.41 mg/dL 5 months before admission (Table 1). 

### Diagnostic evaluation 

Upon arrival, the patient was afebrile at 37 °C, his blood pressure was 140/80 mmHg, pulse 80 beats/minute, and oxygen saturation 98% in room air. Clinical examination was overall unremarkable, except for mild diffuse tenderness upon palpation of the abdomen. Due to his symptoms, he was initially evaluated by the surgeons, where a nasogastric tube was placed, rendering bile content. Laboratory routine blood tests showed elevated SCr (11.77 mg/dL), urea (237 mg/dL), and potassium (6.6 mEq/L) as well as leukocytosis (13.200/μL), with increased polymorphonuclears and high C-reactive protein (CRP) levels (95.6 mg/L) (Table 1). The analysis of arterial blood gases showed pH = 7.26, pCO_2_ = 21 mmHg, pO_2_ = 106 mmHg, HCO_3_^–^ = 9.2 mEq/L, and lactates = 45 mg/dL. A kidney ultrasound showed kidneys’ size within normal with increased renal cortex echogenicity, preservation of corticomedullary definition, and no evidence of lithiasis or obstruction. A urine catheter was inserted without providing any urine. 

### Hospital course 

Based on clinical and laboratory findings, the patient was admitted at the Nephrology Department. Intravenous fluid therapy administered to the patient during the first day consisted of 1,000 mL dextrose-containing (D/W) solution (5%) enriched with 12 units of insulin for correction of hyperkalemia, 100 mL of NaHCO_3_ solution (94 mEq) for correction of metabolic acidosis as well as 500 mL of normal saline (N/S), to rule out volume depletion as a probable cause of prerenal AKI. Following absence of conversion of oligoanuric to nonoliguric AKI, intravenous furosemide was initiated at a dose of 60 mg twice in a day (t.i.d.), and the patient was monitored for changes in urine flow rate. Furthermore, a broad-spectrum intravenous antibiotic regimen with vancomycin 1 g as a loading dose (and then 500 mg every other day) and ciprofloxacin 200 mg t.i.d. was initiated. Metformin, olmesartan, amlodipine, hydrochlorothiazide, acetylsalicylic acid, atorvastatin, and finasteride were discontinued. Due to persistence of anuria, central venous catheter (CVC) was placed, and an urgent hemodialysis session was performed within a few hours. 

For the first 3 days, the patient continued to have urine volumes of less than 100 mL/24h and persistently elevated creatinine (11.94 mg/dL on day 2 and 11.25 mg/dL on day 3) and urea (248 mg/dL and 176 mg/dL, respectively). Computed tomography (CT) of the abdomen was performed on day 2 and showed mild perinephric fat stranding, absence of lymph node enlargement, or free intra- or retroperitoneal fluid collection. Technetium-99m-diethylenetriaminepentacetate acid (DTPA) renal scintigraphy also performed on day 2 revealed adequate perfusion of both kidneys, without signs of vascular occlusive event or obstructive uropathy, but with significant severe bilateral tubular dysfunction. 

On day 4, diuresis was started (900 mL/24h) and gradually increased. Urinalysis was positive for presence of glucose (50 mg/dL), protein (100 mg/dL), and significant amount of hemoglobin (+++), while sediment at that day showed over 200 erythrocytes, and 6 – 10 leukocytes per high-power field. A 24-hour urine collection, performed on day 8, showed excretion of creatinine at 859.32 mg/24h and protein at 1.4 g/24h. Urine sediment showed 3 – 5 erythrocytes, and 0 – 2 leukocytes per high-power field. Urine microscopy did not reveal dysmorphic erythrocytes, while no erythrocyte casts were observed. Progressive decline of the hematocrit (from 41 to 29.6%) and platelets (217,000/mm^3^ – 120,000/mm^3^), after ruling out bleeding as a possible cause, was treated with erythropoietin and discontinuation of aspirin. Evaluation of peripheral smear did not reveal schistocytes, and no increase in serum LDH levels were detected; therefore, microangiopathic hemolytic anemia was excluded. 

Despite supportive treatment, kidney function did not show signs of considerable improvement. Despite detailed discussions over the previous history, only on day 8 did the patient remember to report consumption of tea prepared with mountain herbs of unknown origin for ~ 30 days before his symptoms started. As, pre-renal and post-renal causes of AKI were already conclusively excluded and in waiting for the laboratory results for possible intrinsic renal causes, our differential diagnosis included also AIN, and pulse steroid treatment was therefore initiated on day 8 (methylprednisolone 125 mg on the first day and then 62.5 mg o.d. for 5 consecutive days, and 48 mg orally thereafter). 

**Figure 1:**
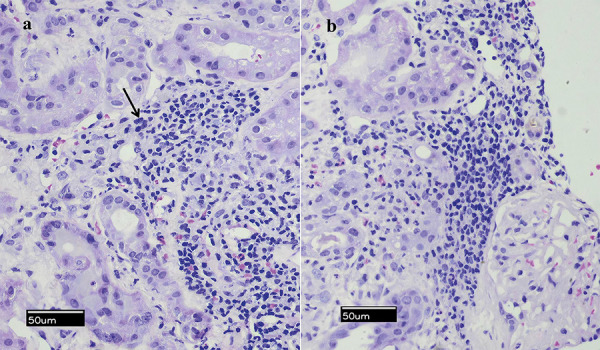
Kidney biopsy revealed presence of (a) moderate to severe tubulointerstitial inflammatory infiltrates mainly composed of lymphocytes and eosinophils with acidophilic (reddish) cytoplasm; (b) nonspecific mild mesangial hyperplasia of glomeruli (hematoxylin & eosin stain; original magnification × 200).

Over the next days, serum immunology testing (IgG, IgA, IgM, C3, C4, ANA, ANCA, anti-dsDNA, anti-GBM, rheumatoid factor, serum protein electrophoresis, and total κ/λ chains) was negative, except for the detection of elevated immunoglobulin-free κ/λ chains. The patient’s blood cultures, urine cultures, and viral serologies (EBV, CMV, HCV, HBV, and HIV) were also negative. Although the patient restored diuresis, he remained dialysis dependent and, thus, an ultrasound-guided kidney biopsy was performed on day 14. We obtained one kidney biopsy cylinder including 17 glomeruli, which was processed for light microscopy, one cylinder including 8 glomeruli, which was processed for immunofluorescence, and a smaller third tissue sample that was later sent for PCR testing. Light microscopy revealed moderate to severe tubulointerstitial inflammatory infiltrates, mainly composed of lymphocytes, neutrophils, and some eosinophils (Figure 1). There was also interstitial edema and tubular atrophy, corresponding to 25 – 30% of the cortical area. Some chronic vascular changes were additionally observed, with hyalinization of some arteriolar walls. The glomeruli showed presence of nonspecific mild mesangial hyperplasia. Segmental necrosis, crescents or abnormalities of the glomerular basement membrane were absent. Immunofluorescence was negative and showed no evidence of immune deposits. The above findings were compatible with AIN, confirming our hypothesis. On this basis, testing for hantavirus and leptospira antigen was available within a few days, showing serum enzyme-linked immunosorbent assays (ELISA) IgM antibodies positive for hantaan with progressive increase in titer in a second sample, from 2.5 to 4.3 (normal values < 1.5). Immunofluorescence of IgG and IgM antibodies for hantavirus as well as serum PCR were negative. The third biopsy sample was sent for PCR analysis for detection of genetic material of hantavirus; however, the sample was inappropriate for testing. 

After the biopsy, the patient was in a stable condition, with his kidney function constantly improving with progressive methylprednisolone tapering, last hemodialysis session at day 18 (10 sessions in total) and SCr = 2.38 mg/dL at day 23. At day 22, the patient underwent a molecular test for SARS-CoV-2 due to presentation of SARS-CoV-2 positive patients and staff of the Nephrology ward, possibly due to intra-hospital transmission during the COVID-19 second wave (November – December 2020). The patient tested positive and, although being totally asymptomatic, he was transferred to the COVID-19 positive wards, where he continued to receive nephrology care for another 14 days. Methylprednisolone, after receiving 32 mg for 20 days, was further tapered, with his kidney function progressively improving. He was discharged with the dosage of 20 mg and instructions to taper by 4 mg every 2 weeks to 12 mg, then by 4 mg every week to a dose of 4 mg, and then by 2 mg every 2 weeks. Steroid treatment had been discontinued after a total of 13 weeks. Regular follow-up visits were scheduled in our Outpatient Department, with kidney function further improving (SCr of 1.76 mg/dL 1 month after discharge and 1.44 mg/dL 6 months after discharge). 

## Discussion 

AKI is a broad clinical syndrome encompassing various etiologies. In our case, the 64-year-old farmer presented with severe dialysis-dependent AKI superimposed on CKD stage 3. After careful investigation of the cause of kidney failure, and despite absence of compatible clinical symptoms, the diagnosis of hantavirus-associated AIN was made. 

Hantavirus is an uncommon cause of infection-related AIN [3]. Hantaviruses (genus Hantavirus, Bunyavirales order) are transmitted to humans through inhalation of the aerosolized excreta of infected rodents, belonging to the Murinae, Arvicolinae, and Sigmodontinae subfamilies of the Muridae family [4]. Farmers, shepherds, woodcutters, forestry workers, animal trappers, mammalogists, hunters, and military personnel are considered high-risk groups [4]. Three strains of hantaviruses are known to cause HFRS in Europe: Puumala, Dobrava, and Saaremaa, with the first two being the most prevalent [4]. The incubation period is 2 – 4 weeks [4]. Puumala, carried by the red bank vole (*Clethrionomys glareolus*), causes nephropathia epidemica (NE), a milder form of HFRS [4], while Dobrava, carried by the yellow-necked mouse (*Apodemus flavicollis*), causes severe kidney injury, mostly in the Balkans [4]. The 10-year prognosis is more favorable for Puumala infection, although some patients develop hypertension [5, 6]. 

HFRS represents a systemic inflammatory response syndrome, with a multifactorial pathogenesis [7], including increased cleavage of high molecular weight kininogen, higher enzymatic activities of FXIIa/kallikrein, and increased liberation of bradykinin from endothelial cells, resulting in overactivation of the kallikrein-kinin system and increased endothelial cell permeability [8]. Compromised barrier function and increased capillary leakage may be associated with proteinuria [9], while glycosuria can result from proximal tubular damage, whose severity can vary from mild tubular dysfunction to disturbances typical of Fanconi syndrome [10]; both features were present in our patient and can be attributed to hantavirus-associated kidney injury with glomerular and tubular involvement. Clinical symptoms of typical HFRS include fever, myalgias, headache, abdominal pain, vomiting, and hemorrhagic complications due to thrombocytopenia (gastrointestinal bleeding, hematuria), oliguria, and hypotensive shock [1, 2, 4, 7]. The diagnosis of acute hantavirus infection is based on immunofluorescence and ELISA methods [11]. Virus-specific IgM can be detected even in the first days of the disease, when IgG antibodies may be also present [11]. 

While HFRS is endemic on the Balkan Peninsula, most cases in Greece are sporadic, with the first case appearing in 1982; however, epidemic outbreaks have also been described [11]. The predominant hantavirus is Dobrava. Pindos and Rhodope (northwest and northeast parts of Greece) are considered to be hyperendemic areas, with 80% of total HFRS cases described in these two regions [12]. The clinical manifestations in Greece range from mild or moderate to severe or fatal illness [11]. However, Elisaf et al. [12] suggested that mild cases (without hemorrhagic manifestations) or asymptomatic cases may be more common than severe types of disease, and that 5 – 10% of cases are not detected. 

In the kidneys, the capillary leakage results in transient proteinuria, hematuria, and kidney failure [13]. Typical kidney biopsy findings include acute tubulointerstitial nephritis with interstitial edema and inflammatory cell infiltrations; tubular epithelial and luminal alterations and slight glomerular mesangial changes are present in 25% of specimens [14]. Interstitial hemorrhage is a characteristic finding that may be missed because of the absence of medullary sampling [15]. Our patient presented with minimal clinical symptoms despite the presence of severe AKI, with a biopsy compatible with the severe tubulointerstitial nephritis observed with HFRS. As typically renal biopsies target the renal cortex, our samples also did not include sufficient sampling of renal medulla; thus, interstitial hemorrhage could have been missed. 

Treatment of HFRS consists of supportive care mainly including maintenance of electrolyte and fluid homeostasis and correction of hematologic abnormalities; dialysis treatment may be required in some cases where conservative treatment fails, with compete recovery of kidney function most of the time [4, 7]. Additionally, ribavirin has been associated with a seven-fold decrease of mortality and a significant reduction in the risk of entering the oliguric phase and experiencing hemorrhage [16]. Corticosteroid use for treatment of hantavirus infections is common, and successful treatment for HPS [17, 18], severe thrombocytopenia [19] as well as recovery of kidney function, have been reported [20]. However, the use of corticosteroids for treatment of HFRS is limited to observational studies, and no evidence from randomized trials is available. In our case, steroid treatment was associated with complete recovery of kidney function at 6 months after discharge. 

COVID-19, declared as a pandemic by WHO in March 2020, has affected more than 270 million individuals and caused more than 5.4 million deaths worldwide [21]. An increase in mortality was observed in Greece during November-December 2020 when the country faced a particularly strong second wave [22]. The risk of severe illness and mortality in COVID-19 increases with older age, male sex, and comorbidities, including hypertension, diabetes mellitus, cardiovascular disease, and CKD [23], and may be further increased by intra-hospital transmission during an extensive outbreak [24]. Thus, our patient faced a high mortality risk after COVID-19 infection, due to his age and the presence of several cardiovascular risk factors. However, despite his long hospital stay and severely impaired kidney function, which could have been further deteriorated by SARS-CoV-2 co-infection, the patient was finally discharged with moderate kidney failure after receiving steroid treatment. Use of steroids has received strong recommendation in practice guidelines for severe illness in COVID-19 [25]. Steroid treatment, already initiated in our patient, could have helped prevent adverse outcomes. 

To conclude, our patient’s case highlights the fact that hantavirus nephropathy in countries such Greece may present clinically as AKI of unknown origin with minimal symptoms and signs of infection and no hemorrhagic manifestations. As such, hantavirus-associated acute tubulointerstitial nephritis should remain in the differential diagnosis of such patients, especially if common causes of AKI are precluded. In such cases, timely testing for hantavirus antibodies and kidney biopsy could lead to the diagnostic answer, and appropriate treatment can help towards recovery of kidney function. 

## Funding 

This paper was not supported by any source and represents an original effort of the authors. 

## Conflict of interest 

None. 

**Table 1.** Laboratory investigation results.

Variable	18 months before	5 months before	Day 1 admission	Day 4	Day 8	Day 9	Day 11	Day 15	Day 18	1 month later	4 months later	6 months later	Normal value or range
White-cell count (10^3^/μL)	–	–	13.2	6.4	7.7	10.2	11.9	15.5	16	13.9	9.8	6.9	3.8 – 10.5
Hematocrit / Hemoglobin (%, g/dL)	–	–	13.7/ 41	10.3/ 29.6	10.3/ 30	10.4/ 30.1	10.4/ 29.9	11.1/ 32.1	12.5/ 36.5	12.7/ 37.3	13.3/ 40.7	14.2/ 43.2	12.0 – 16.0/ 37.0 – 47.0
Platelets (10^3^/μL)	–	–	217	120	201	215	207	78	98	215	199	215	150 – 450
Total serum proteins (g/dL)	–	–	7.3	6.5	5.9	6	6.1	4.1	6.2	6.6	–	4.7	6.6 – 8.3
Serum albumin (g/dL)	–	–	4.5	4.1	3.7	3.6	3.8	4.1	3.9	4.1	–	3.5	3.4 – 5.2
Serum glucose(mg/dL)		–	137	120	168	200	208	220	179	147	–	–	70 – 125
Serum urea (mg/dL)	33	50	237	137	155	113	144	156	168	99	66.3	49.5	15 – 43
Serum creatinine (mg/dL)	1.29	1.41	11.77	9.92	13.65	10.7	8.59	4.94	3.93	1.76	1.70	1.44	0.66 – 1.1
Serum potassium (mEq/L)	–	–	6.6	4.7	4.7	4.9	4.9	4.8	4.4	5.2	4.49	5.2	3.5 – 5.1
Serum sodium (mEq/L)	–	–	151	132	138	138	140	139	134	138	143	–	136 – 145
Serum calcium (mg/dL)	–	–	10.2	8	7.4	8.2	8.2	8.4	7.7	9.9	9.7	9.5	8.5 – 10.5
Serum phosphorus (mg/dL)	–	–	5.1	6.1	5.9	4	5.6	6.4	4.3	4.8	4.1	–	2.5 – 4.5
Serum magnesium (mg/dL)	–	–	2.38	–	–	–	–	1.65	–	1.58	–	–	1.9 – 2.5
Serum uric acid (mg/dL)	–	–	11.2	5.1	6.2	5	–	6	4.8	5.6	7.53	6.9	2.6 – 6.6
Serum CRP (mg/L)	–	–	95.6	25.6	17.6	17	9.1	15.8	3.8	1.9	–	–	< 6
ESR (mm/h)	–	–	–	–	36	–	–	–	–	17	–	–	0 – 20
LDH (U/L)	–	–	282	280	244	241	245	245	243	–	–	–	100 – 248
Hb-A1c (%)	–	–	7.2	–	–	–	–	–	–	6.1	–	–	4 – 6
Parathyroid hormone (pg/mL)	–	–	27.8	–	–	–	–	189.8	–	27	42.7	–	12 – 88
24-hour urine collections													
Urine volume	–	–	–	900	2,100	2,200	2,200	–	3,500	–	3,500	3,800	1,000 – 2,500 mL/24h
Urine creatinine	–	–	–	–	859.32		898.9	–	1593	–	1.41	–	14 – 26 mg/24h/kg
Urine urea	–	–	–	–	6.2	–	7.9	–	33.9	–	–	–	20 – 35 g/24h
Urine total protein	–	–	–	–	1,400	–	259	–	248	–	67	129	< 150 mg/24h
Urine sodium	–	–	–	–	185	–	205	–	182	–	133	160	50 – 200 mEq/24h
Urine potassium	–	–	–	–	19	–	29	–	67	–	70	66	30 – 90 mEq/24h
Urine glucose	–	–	–	–	1,638	–	5,236	–	46,830	–	–	–	< 500 mg/24h
Urinalysis													
Urine protein (mg/dL)	Negative	Negative	–	100	–	–	–	–	7.1	Negative	Negative	Negative	Negative
Urine glucose (mg/dL)	Negative	Negative	–	50	200	–	–	–	1,338	Negative	Negative	Negative	Negative
Urine hemoglobin	Negative	Negative	–	+++	+	–	–	–	–	Negative	Negative	Negative	Negative
Urine erythrocytes	–	–	–	> 200	3 – 5	–	–	–	–	0 – 2	1 – 2	0 – 2	3 – 5/hfp
Urine leukocytes	–	–	–	6 – 10	0 – 2	–	–	–	–	0 – 2	1 – 2	0 – 2	0 – 5/hfp

CRP = C-reactive protein; ESR = erythrocyte sedimentation rate; LDH = lactate dehydrogenase; Hb-A1c = glycated hemoglobin A1c; hpf = high power field.
